# The heart of palliative care is relational: a scoping review of the ethics of care in palliative medicine

**DOI:** 10.1186/s12904-025-01784-5

**Published:** 2025-05-26

**Authors:** Sophie Bertaud, Dominic Wilkinson, Maureen Kelley

**Affiliations:** 1 Ethox Centre, Nuffield Department of Population Health, University of Oxford , Big Data Institute, Old Road Campus, OX3 7LF Oxford, UK; 2 Louis Dundas Centre for Children’s Palliative Care, Great Ormond Street Hospital for Children, London, UK; 3 Uehiro Oxford Institute, University of Oxford, Oxford, UK; 4 John Radcliffe Hospital, Oxford, UK; 5 Department of Internal Medicine, Wake Forest University School of Medicine, Winston- Salem, USA; 6 Center for Bioethics, Health & Society, Wake Forest University, Winston-Salem, USA

**Keywords:** Palliative care, Hospice and palliative care nursing, Bioethics, Ethics, clinical

## Abstract

**Background:**

Palliative care, perhaps more than any subspecialty in healthcare, is deeply relational and engages patients and families at times of great vulnerability. Ethics of care, or relational ethics, developed through contributions from feminist ethics, offers conceptual tools and ways of thinking that seem especially suited to palliative care practice.

**Aim:**

To identify and describe studies and theoretical analyses applying the ethics of care to palliative care (both adult and paediatric), specifically, its use to guide and improve practice and education for palliative care practitioners.

**Design:**

We conducted a scoping review of six databases covering clinical, social science and normative ethics scholarship and conducted a thematic analysis of the findings and ethical discussions or arguments.

**Data sources:**

Databases searched included PubMed, CINAHL, PsychINFO, EMBASE, Web of Science and Philosopher’s Index from 1982 to November 2024.

**Results:**

30 publications meeting our inclusion criteria were identified. Major themes reflected the relational obligations, attributes and character traits ideally developed in palliative care providers in their work and relationships with patients and families, including responsiveness, connectedness and hope, as well as in caring for ourselves and each other on palliative care teams. An emerging literature recognises the special guidance for palliative care for children.

**Conclusions:**

Clinical and ethical scholarship in palliative care reveals a valuable but still underexplored connection between the ethical commitments within the ethics of care tradition and palliative care training and practice. Ethics of care addresses important gaps in training, particularly having to do with practitioners’ relationships and ways of being with patients, families, colleagues and themselves.

**Supplementary Information:**

The online version contains supplementary material available at 10.1186/s12904-025-01784-5.

## Background

Relational ethics is an attempt to ground healthcare in our commitments to each other. While theoretical positions vary, the ethics of care tradition is guided by shared recognition that human beings are interdependent, and need respect, protection, and care [1]. Understanding how our embodied interdependence informs our ethical obligations to one another and ways of being and doing, requires greater attention to relationality, contextuality, vulnerability, and attention to power [2]. According to this approach, an ethical approach to decision making requires attention to the issues of power that are inherent in health care decisions and interventions, the vulnerability and dependency of those that are ill, and the role of emotion in authentic human experience [3].

Palliative care seeks to improve quality of life by addressing the physical, psychological, social and spiritual needs of people facing life-threatening illness, and their families [4]. Palliative care is arguably a medical specialty grounded in the principle that a positive relationship between patients and their care team is essential to the delivery of effective palliative care [5]. Ethics of care is a normative ethical theory which developed through contributions from feminist ethics in the 1980s and argues for the moral preferability of a relational care perspective, which focuses on the needs of those that one cares for in relational contexts, over and above abstract, universal principles [6]. In the same way that palliative care recognises the importance of the relationship between practitioners and patients, ethics of care emphasises that caring well both requires, and is an expression of, a caring relationship. It requires one to care about the person you care for, to engage with their will and to pay close attention to the context and the particulars of the individual person [7]. Critics of ethics of care argue that it valorises femininity and risks reinforcing gendered stereotypes of caregivers which might expose those who deliver care to exploitation or a loss of integrity [7]. Furthermore, because it rejects abstract principles, care ethics has been accused of being ambiguous, and for failing to offer concrete guidance for ethical action [8].

This scoping review explores what ethics of care may have to offer to support the practice of adult and especially paediatric palliative care. Paediatric Palliative Care is now established as an important subspecialty within paediatrics which focuses on the care of children and young people with life-limiting conditions, but which also importantly encompasses the care and support of their parents/carers and wider family. Within the paediatric setting particularly, nurturing relationships are pivotal to providing care and hence this review sought to interrogate the potential application of ethics of care to this field.

## Methods

This review is part of a larger normative and empirical ethics study on antenatal palliative care ethics. As such, we were interested in prior applications of ethics of care or relational ethics to palliative care in general, as well as to paediatric palliative care specifically. We sought to identify normative or theoretical publications relating to the ethics of care and palliative care. There are recognised challenges in reviewing both normative and empirical literature in a single review but a systematic scoping review approach can offer the means of identifying key ideas in particular areas of bioethics and critically engaging with them [9]. We utilised the first 5 stages of the 6-stage process as proposed by Arksey and O’Malley [10] as follows:

1. Identifying the research question;
2. Identifying relevant studies;
3. Study selection;
4. Charting the data;
5. Collating, summarizing, and reporting the results; and.
6. Consultation with practitioners/consumers.

The sixth stage—consultation with practitioners/consumers—is an optional stage. As this scoping review is part of a larger project, involving patient and public involvement representatives and collaboration with key stakeholders in paediatric palliative care, the sixth stage is an ongoing element of the wider project and did not directly inform this review. A PRISMA for Scoping Reviews (PRISMA-ScR) checklist is provided Supplementary File 1.

### Stage 1: identifying the research question

The research question was developed by SB, DW and MK following informal discussions with both palliative care practitioners and bereaved parents. The research question was defined as follows: how does ethics of care apply to palliative care (both adult and paediatric)?

In answering this question, this scoping review aimed to collate evidence relevant to the following secondary research questions:

- How can ethics of care illuminate the key activities of palliative care practitioners?
- How can ethics of care guide and improve palliative care practice?
- What is the role of ethics of care in palliative care education and training?

### Stage 2: identifying relevant studies

We searched six databases (PubMed, CINAHL, PsychINFO, EMBASE, Web of Science and Philosopher’s Index) to identify relevant studies published between 1982 (when care ethics was first explicitly articulated by Carol Gilligan [11]) and November 2024. The search strategy was defined with the support of a librarian from the Bodleian Health Care Libraries. Several search strategies, using a variety of search terms, were tested to balance scope and ensure target test articles were captured before settling on the strategy outlined in Table 1. The search was conducted in February 2024 and updated in November 2024.

**Table 1:** Search strategy

Query 1		Query 2
“ethics of care”		“palliative care”
OR		**OR**
“ethic of care”	**AND**	“supportive care”
OR		**OR**
“relational ethics”		“end of life care”
OR		**OR**
“feminist ethic*”		eol
		**OR**
		eolc
		**OR**
		hospice
		**OR**
		“life-limiting condition”
		**OR**
		llc
		**OR**
		“life-threatening condition”

### Stage 3: study selection

**Fig. 1:**
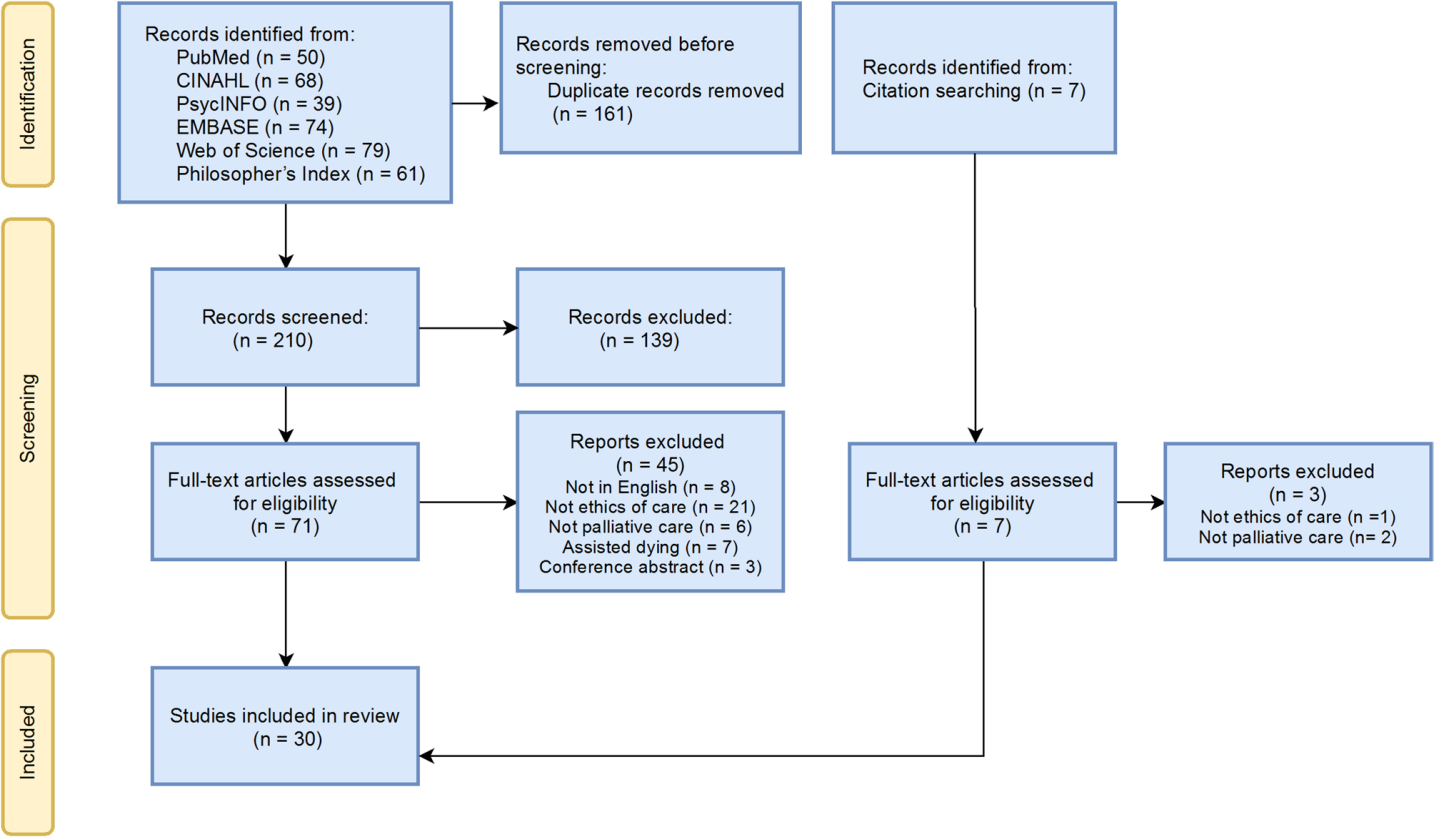
PRISMA flowchart

After duplicate records were removed 210 study abstracts were screened by SB of which 139 studies were excluded as they did not relate to the research question. 78 full text-articles were assessed for eligibility, 7 of which had been identified by additional citation searching. Reports were excluded if they did not relate to both ethics of care and palliative care, if the report was not available in English or constituted a conference abstract. 7 papers were retrieved with a focus on assisted dying, one of which also did not relate to ethics of care; the remaining 6 papers were excluded as they primarily considered the legalisation of assisted dying in Canada and their focus was felt to be too specific to answer the research question. Any reports where the decision of whether to include the study was unclear were discussed with a second reviewer, MK. A total of 30 studies were included in the final review. Figure 1 shows the PRISMA flowchart for this literature search.

### Stage 4: charting the data

Data from the 30 included studies was charted using a spreadsheet in Microsoft Excel based on the following study characteristics:

- Author.
- Year of Publication.
- Country of origin.
- Title.
- Publication type (journal article, thesis).
- Focus on adult or paediatric palliative care.
- Study aim.
- Main arguments.
- Study recommendations.

Table 2 Lists the studies included. A sample of the papers were also reviewed by a second reviewer, MK.

**Table 2:** Studies included in the review

Author	Year	Country of origin	Title	Type	Focus
Aramesh [12]	2017	Iran	Compassion as the reunion of feminine and masculine virtues in medicine	Conceptual paper	Role of compassion in cases of medical futility.
Bergman et al. [13]	2024	Netherlands	A care ethics approach to a reduced ability to eat	Conceptual paper	A normative analysis, using an ethics of care approach, informing care for patients with reduced ability to eat, and their families.
Berlin [14]	2022	USA	Close Encounters of the First Kind: An Interdisciplinary Ethics of Care Approach Mitigates Moral Injury and Family Division in the Midst of Covid-19	Case narrative	A palliative care physician applies an interdisciplinary ethics of care model to discuss the case.
Borgstrom and Walter [15]	2015	UK	Choice and compassion at the end of life: A critical analysis of recent English policy discourse	Conceptual paper	Analysis of UK policy discourse on end-of-life care focusing on the concepts of choice and compassion
Branch [16]	2015	USA	The Ethics of Patient Care	Commentary	Insights from a geriatric physician discussing care ethics.
De Panfilis et al. [17]	2019	Italy	“I go into crisis when…”: ethics of care and moral dilemmas in palliative care	Empirical study	Qualitative interviews with health care professionals working in palliative care.
De Vries & Leget [18]	2012	Netherlands	Ethical dilemmas in Elderly Cancer Patients: A Perspective From the Ethics of Care	Conceptual paper	Two cases of elderly cancer patients discussed from the perspectives of principlism and ethics of care
Drongowski [19]	1994	USA	Beyond ‘justice vs care’: Can the ethic of care coordinate with the ethic of justice to support palliative care?	Conceptual MA Thesis	Conceptual discussion of justice and ethics of care as applied to palliative care policy
Gilbert and Lillekroken [20]	2024	Norway	Nurses’ perceptions of how their professional autonomy influences the moral dimension of end-of-life care to nursing home residents– a qualitative study	Empirical study	Qualitative interviews with nurses providing end of life care in nursing homes using an ethics of care framework
Grealish [21]	1997	Australia	Ethics. Beyond Hippocrates: ethics in palliative care	Conceptual paper	Ethics of care discussed as a framework for ethical analysis of nursing practice with a focus on palliative care nurses
Guité-Verret et al. [22]	2023	Canada	Intentional presence and the accompaniment of dying patients	Conceptual paper	Discussion of the presence of clinicians working in end of life care settings to inform relational ethics theory
Ho and Lin [23]	2020	Hong Kong	How the COVID-19 Pandemic Could Reshape Palliative Care Into High-Tech and High-Touch Care: An Ethics of Care Perspective	Editorial	Discusses the rise of digital health in palliative care using an ethics of care perspective
Hudson and Wright [24]	2019	Canada	Towards a Guiding Framework for Prison Palliative Care Nursing Ethics	Conceptual paper	Exploration of a relational ethics approach to palliative care nursing in prisons
Leung and Esplen [25]	2008	Canada	Alleviating existential distress of cancer patients: can relational ethics guide clinicians?	Review	Reviews empirical research to explore the experiences of clinicians working with cancer patients with existential issues. A relational ethics lens is used in the analysis.
Morberg Jämterud [26]	2022	Sweden	Acknowledging vulnerability in ethics of palliative care - A feminist ethics approach	Conceptual paper	A feminist ethics perspective on vulnerability in palliative care
Nogueira et al. [27]	2023	Brazil	Ethical dilemmas at the end of life: a reflection from the Philosophical Perspective of Luigina Mortari	Conceptual paper	Reflection on ethical dilemmas in caring for patients at the end of life using an ethics of care perspective
Olsman et al. [28]	2016	Netherlands	Solicitude: balancing compassion and empowerment in a relational ethics of hope—an empirical-ethical study in palliative care	Empirical study	Qualitative interviews with palliative care patients on hope are used to describe a relational ethics of hope
Olthuis and Dekkers [29]	2003	Netherlands	Professional Competence and Palliative Care: An Ethical Perspective	Conceptual paper	Examination of professional competence of physicians in palliative care from a virtue ethics and an ethics of care perspective
Pergert and Lützén [30]	2012	Sweden	Balancing truth-telling in the preservation of hope: a relational ethics approach	Conceptual paper	Exploration of the relationship between the concepts of truth-telling and hope from a relational ethics approach. Focus on the context of palliative care
Peter and Liaschenko [31]	2013	Canada & USA	Moral distress re-examined: A feminist interpretation of nurses’ identities, relationships, and responsibilities	Conceptual paper	Moral distress in nursing practice is examined from a feminist ethics perspective. Focus on moral distress as a response to aggressive care at end-of-life.
Ramvi and Ueland [32]	2019	Norway	Between the patient and the next of kin in end-of-life care: A critical study based on feminist theory	Empirical study	Focus group interviews with nurses to explore ethical challenges in relation to next of kin in end-of-life care
Smith and Stajduhar [33]	2024	Canada	Using relational ethics to approach equity in palliative care	Conceptual paper	Exploration of relational ethics as a way to approach equity in palliative care.
Wasson [34]	1998	UK	The Ethics of Care or the Ethics of Justice? A Middle Way	Conceptual PhD Thesis	Critical analysis of the tension between ethics of justice and the ethics of care. Uses several palliative care examples
Wright D et al. [35]	2009	Canada	Human relationships at the end of life: an ethical ontology for practice	Conceptual paper	Discussion of relational ethics as a framework to guide palliative nursing practice. Use of mini case narratives
**Papers with a paediatric focus**					
Brierley and Larcher [36]	2011	UK	Cui bono? Can feminist ethics show a path in complex decision-making where ‘classical’ theories cannot?	Case discussion	Ethical discussion of a case of a 6-year-old with a fatal brainstem tumour where feminist ethics proved useful.
Moreton [37]	2019	UK	Reflecting on ‘Hannah’s Choice’: Using the Ethics of Care to Justify Child Participation in End of Life Decision-Making	Conceptual paper	Case of a 12-year-old who refused a heart transplant discussed through the parent’s narrative account and using an ethics of care framework.
Moreton [38]	2017	UK	The ethics of care and healthcare decision-making involving children in mid-childhood	Conceptual PhD thesis.	Presents a normative framework based on ethics of care to aid healthcare decision-making for children in mid-childhood.
Neefjes [39]	2023	UK	Parental Ethical Decision Making and Implications for Advance Care Planning: A Systematic Review and Secondary Analysis of Qualitative Literature from England and Wales, Germany, and the Netherlands	Systematic review and secondary qualitative analysis	Reviews qualitative literature on parental decision-making for children with life-limiting conditions to assess whether it was consistent with ethics of care.
Walter and Ross [40]	2014	USA	Relational autonomy: Moving beyond the limits of isolated individualism	Conceptual paper	Explorations of individualistic and relational models of autonomy in the context of decision-making in paediatric palliative care.
Whitty-Rogers et al. [41]	2009	Canada	Working with Children in End-of-Life Decision Making	Conceptual paper	Case studies are used to explore the complexity of ethical challenges facing nurses in end-of-life care with children and families. A relational ethics framework is used.

### Stage 5: collating, summarizing, and reporting the results

A thematic content analysis approach [42] was used to identify, collate and summarise the data according to central ethical themes. We applied a conceptual ethics framework with a list of prompts and questions designed to help draw out the key ideas at the intersection between ethics of care and palliative care. Conceptual codes were developed from ethics of care theory and a list of tentative codes was created and modified iteratively. Our approach is detailed in Supplementary File 2.

Once initial coding was completed, the team met to review how the codes related to each other and to identify patterns among them, leading to the creation of the themes and sub-themes outlined below. Throughout the process, the reviewers made notes and discussed ideas within the research team.

## Results

The papers included in this review came from a wide range of countries, including China, Brazil and Iran, although were predominantly from Western Europe and North America. All considered palliative care as delivered by medical, nursing and allied health professionals, although many papers emphasised the special role that the nursing profession can play in delivering palliative care with an ethics of care focus. Given that care ethics emerged from nursing ethics practice and scholarship, it is not surprising that authors reinforced this connection, as stated by Grealish “palliative care nurses are well placed to promote and research the ethic of care” [21].

The overarching theme emerging from the literature was the interconnectedness of patients and health care providers in palliative care. Ethics of care teaches that the interests of carers and those that are cared-for are importantly intertwined rather than simply competing [43] and this was strongly reflected in our findings. Beneath this over-arching theme of the interconnectedness of patients and healthcare professionals working in palliative care, three key themes were identified: our ways of being with patients and families, our ways of being specifically with paediatric patients and their families, and our ways of being with ourselves and each other on care teams. Within these three themes several sub-themes were developed as outlined below.

A cross cutting finding throughout the data was that both our day-to-day practice in palliative care and the education and training of all health care professionals who encounter palliative care patients, can be richly informed by an ethics of care.

### Ways of being with patients and families

The majority of the papers in the review discussed the importance of ways of being with, and related to, patients receiving palliative care and their families. This included the emotional dimensions of being present during a transition that everyone will navigate in their own way. Within these papers ethics of care was able to illuminate the daily practice of care giving and the skills it requires, such as attentiveness, and highlight areas where care could be improved or could respond to new challenges or circumstances such as the Covid-19 pandemic or the integration of digital health into palliative care. With this focus on relationality, three key sub-themes emerged: being with responsiveness and respect, being interconnected with empathy, and being with hope.

#### Being with responsiveness and respect

Concepts central to the ethics of care such as attentiveness, responsiveness and respect were highlighted as key skills necessary for high quality palliative care that meets the needs of palliative care patients and their families. Palliative care is at its best when it is carefully tailored to the needs of the patient, and able to respond (and to adapt) to the shifting needs of an individual over their life course. One of the strengths of an ethics of care was thought to be the way it encourages a delicate balance between recognising and being attentive to patients’ and families’ immense vulnerability at the end of life while remaining responsive to their wishes and respectful of their autonomy in health decision-making. Morberg Jämterud [26] discusses how patients in need of palliative care are often labelled as vulnerable but that by adapting a relational concept, vulnerability and autonomy need not stand in opposition to one another. Fostering relational autonomy requires a trusting interpersonal environment, and nurses are suggested as being particularly suited to facilitating this through continuous interpersonal discussions both with patients, families and within the healthcare team. As highlighted by De Vries and Leget [18]“being attentive and responsive to the patient’s perspective seems to be a major precondition for delivering good quality care attuned to the need, perspective and vulnerable position of patients”.

#### Being interconnected with empathy

Elements of interdependence and connectedness or interconnectedness also featured in many of the papers. Care ethics understands interdependence as a necessary aspect of the human condition; we will all be dependent on others for care at some point in our lives. Interconnectedness is a related concept, whereby we are linked together as people, our interests are aligned, and our well-being depends on each other. When healthcare professionals are able to recognise their shared humanity with their patients and empathise with their experiences, this allows a caring relationship to develop where human suffering is shared, and patients feel truly accompanied, as they approach the end of their life. As Leung and Esplen [25] explain:

*Relational ethics respects the needs for autonomy but also dependence*,* dignity but also connectedness. Only when clinicians recognize their shared mortality*,* can they imagine facilitating a journey of dying that can culminate to a ‘good’ death.*

They argue that to connect to their patients, clinicians are required to develop their moral sensitivity such that they actively engage with a patient’s existential distress, recognise their own vulnerability and embody this sensitivity in their practice. Such a way of being doesn’t necessarily involve ‘doing’ per se and sometimes simply offering mindful accompaniment to a patient may be sufficient. As Guité-Verret et al. highlight, “one’s presence allows one to see and meet the other as a subject similar to oneself [22].” There is a crucially an element of reciprocity in such exchanges; caring moral obligations are based upon a notion of give and take, or two-way exchange. For Guité-Verret et al. “a dynamic of reciprocity is initiated when the clinician witnesses and inherits the work that the patient accomplishes in the face of suffering and death [22].”

The ethics of care tradition also helpfully centres family relationships in healthcare, which is especially important in care at the end of life when shifting from curative interventions to supporting patients and their families in meaningful time together. There may be important decisions affecting others in the family, and health care professionals may be navigating different views about what the patient would have wanted when they are no longer able to voice their wishes or when their wishes are unknown. Several of the papers discussed the strengths of interconnectedness in paediatrics which will be discussed below.

#### Being with hope

For a number of the authors included in this review, being with patients at the end of life also requires allowing a space for hope. Leung & Esplen [25] discuss the positive psychological effects on patients with terminal illness of maintaining hope in their attitudes about end of life, even when beyond curative treatment. A relational care ethics encourages palliative care teams to be aware of how their presence, emotions, tone, and language can either create space for a hopeful and positive attitude toward dying, or undermine it. Leung & Esplen go on to argue that health care professionals play a critical role in creating the emotional space around the end of life and “therefore, clinicians are urged to look beyond dominant discourses about pain and symptom management and consider creating personally meaningful deaths to preserve meaning, purpose and hope” [25].

Pergert and Lützén [30] address the challenges faced by many clinicians of having to balance truth-telling within the caring relationship, but they conclude that “hope must always be inspired”. Instead of focusing on autonomy as the only guiding principle, the authors propose that relational ethics can serve as a meaningful perspective in balancing truth-telling. Hope in palliative care practice has traditionally been framed as a simplistic tension between the physician who tells their patient the truth about their diagnosis and thus respects their autonomy versus the physician who allows their patient to retain false hope about their future. Olsman et al. [28] also address this tension and describe how, in reality, patients will naturally oscillate between feelings of hope and empowerment and feelings of loss of hope and suffering. Sharing hope can be a powerful tool in bonding between specialist physicians and their patients. Olsman et al. [28] draw on relational ethics to offer a theory of hope in which “healthcare professionals sensitively attune to patients’ and family members’ suffering and hope” and are able to balance both these emotions simultaneously within reciprocal caring relationships.

### Ways of being with children and their families

Of the six papers with a paediatric focus, including case reports, shared clinical reflections, and one review, a strong theme emerged in relation to the fit between relational care ethics and the complex decision-making which occurs in paediatric palliative care. All six papers addressed shared decision-making and offered feminist ethics or ethics of care as a means of unravelling the often-complex ethical dilemmas which emerge when difficult decisions are being made about treatment for critically unwell children. Similar concepts to those present in the adult literature of interconnectedness, compassion, respect and autonomy, and the importance of hope were present throughout the paediatric papers.

Ethics of care is presented as particularly helpful for helping professionals to appreciate the ‘intricate and intimate’ [41] network of relationships surrounding a child and including this as a relevant moral consideration when making decisions. Being with children in paediatric palliative care emerges as a practice which necessitates seeing the child as part of a wider network of relationships. Brierley & Larcher [36] emphasise this:

*Reviewing the case of the boy in this light allowed the interpersonal relationships that exist within families and social groups to be accorded primacy. It enabled the setting aside of basic considerations of the child’s autonomy or conflicts between competing duties or rights. When this case was considered in such terms the love inside this*,* or indeed other families*,* was afforded more weight*,* and the child seen in his role as son*,* sibling*,* schoolmate*,* friend and brother.*

Relational ethics can enable health care professionals to recognise and respect a child’s connectedness to those around them, especially their parents. As discussed by Whitty-Rogers et al. [41]: “This approach to ethics reinforces the call to connectiveness and compassion, and a commitment to those in one’s care.” Because a child is more deeply dependent on parents and family than many adult patients, an ethics of care underscores the importance of recognising from the outset that palliative care paediatricians are emotionally caring for the parent(s) as much as for the child. Walter and Ross [40] acknowledge the profound challenge of respecting both parental autonomy and the developing autonomy of the child and argue that a relational model of autonomy in paediatric palliative care “provides clinicians with ethical justification for directly engaging families in difficult conversations that acknowledge emotions and for offering parents guidance on the breadth of decisions that can express their love for their child”.

Furthermore, several of the papers suggest the role of ethics of care in helping to give a voice to the child themselves, by allowing professionals to tune in to the realities of the child’s life. As Whitty Rogers et al. [41] suggest “To learn how children think, nurses must first listen to children to be able to relate to them. Hearing children’s voices fosters their choice.” Moreton [37, 38] argues that the ethics of care is preferable to other normative ethical approaches, such as those which focus on traditional principles in biomedical ethics, for allowing care which reflects the reality of most children’s lives and allows them to be involved in decision-making. Being attuned to family relationships can help support trusting relationships with families and enhance decision-making, particularly in ethically complex situations. For example, as an adolescent in cancer treatment transitions to palliative care, an ethics of care may draw attention to the patient’s dynamic with protective parents through a long and difficult treatment course and signal the patient’s need to voice their feelings and wishes in transitioning to symptom-focused care.

The paediatric papers frequently touched on how difficult it can be for both parents, and health care professionals themselves, to give up on the hope for a child’s survival and how this frequently acts as a barrier to engaging families in conversations about palliative care. Palliative care practitioners, particularly nurses, may find themselves at the bedside at a quiet moment of the night when a parent or a child wants to talk. Whitty Rogers et al. [41] emphasise what a ‘powerful position of trust’ this is, and how crucially being honest and open with children instils hope and provides support to them.

### Ways of being with ourselves

Alongside the importance of being with patients and families in palliative care practice, reviewed papers emphasised the importance of paying close attention to how as health care practitioners we consider self-care and care for each other given the especially close emotional connections we have with patients and families and the emotional intensity of the work. Particularly in the palliative care space, patients and their care providers cannot help but share the distress that patients and families experience in this deeply relational work. Two sub-themes emerged under this concept: caring for ourselves and each other, and training for this work.

#### Caring for ourselves and each other

Throughout many of the included papers, an ethics of care approach to palliative care practice is posited as being able to better meet patients’ and families’ needs, but also to have an impact on the well-being of clinicians. Care ethics offers a lens with which to reflect on one’s practice which may help practitioners to engage in deeper reflection and understanding of the moral dimension of their work, especially when caring for patients at the end of their lives. Gilbert and Lillekroken [20] reflect on how in the care provided to nursing home residents in their final days “care ethics can help nurses see and understand the moral dimension of end-of-life care”. Similarly Leung and Esplen [25] argue that an approach to care which is informed by relational ethics may lead to greater work satisfaction for clinicians. Nogueira [27] notes that through a commitment to deeper reflection and understanding of the moral nature of their work, professionals enhance the quality of a patient’s life but also mitigate potential ethical tensions in their caregiving practices. A particularly poignant quote from Branch [16] below, again emphasises the impact that a caring approach can have on a professional’s well-being and how it can connect us with each other within palliative care practice:

*But to feel part of a community that values delivering the very best care*,* working with other caregivers in this endeavor*,* and honing and practicing one’s skills because they are morally meaningful can make doctoring deeply satisfying.*

Conversely the potential for moral distress associated with palliative care practice is well appreciated, and an ethics of care helps us recognise how distress may in some cases be exacerbated by the deeply personal and relational nature of the work. Peter and Liaschenko [31] observe that “moral distress can be interpreted as a response to constraints, or threatened constraints, to the moral identities, relationships, and responsibilities of nurses and other health professionals.” In care at the end of life, the unrelenting course of a terminal disease alongside scarce resources and time, can place limits on what we would ideally hope to do for patients, leaving team members with the distressing feeling that they could not do enough. Ramvi and Ueland [32] discuss how professional relationships with a patient’s family can be a particularly difficult source of moral distress, as the next of kin is often simultaneously someone who needs care and someone who gives care to the patient. They note that for nurses, in particular, it can be “emotionally difficult to contain the anger and frustration expressed by next of kin and at the same time obey orders from them as if they were colleagues”.

#### Training for this work

This collection of papers emphasised the highly intense and emotionally demanding nature of palliative care practice for health care professionals as well as the importance of providing targeted education and training in order to equip staff with the necessary tools to practice in this space. A capacity for self-awareness and self-reflection was particularly emphasised as one of the requisite skills for a palliative care practitioner. Traditional models of medical education have perhaps failed to support clinicians in embracing their own vulnerability and here ethics of care helps to elucidate the importance of this capacity in order to help palliative care professionals to practice with compassion for both their patients, and themselves. As Morberg Jämterud [26] explains:

*To acknowledge vulnerability as a shared life condition one needs training in order to neither be overwhelmed by one’s own vulnerability*,* nor become invulnerable when facing vulnerability in others.*

De Panfilis et al. [17] conducted interviews with both doctors and nurses practicing in palliative care and found that ethics of care emerged as a theoretical framework that best reflected the belief systems of healthcare professionals, especially those assisting patients with palliative care needs. They argue that ethics of care is an appropriate framework for ethical training as it underscores the importance of intensifying relationships and enhancing empathetic involvement and thus allows the values of both patients and professionals to come to light through the relationship of care. Olthuis and Dekkers [29] point out that these intense human interactions seen in palliative care may be able to offer valuable insights across medical training curricula more broadly, with the potential to “improve the ethical culture of health care as a whole”.

Training professionals to work compassionately in palliative care, however, requires that adequate support is in place to help alleviate the moral distress potentially associated with the role. Ramvi and Ueland [32] emphasise “the significance of having a manager that may facilitate systematic supervision where ethical reflections and awareness of nurses’ ‘use of self’ in their practice is in focus.” Perhaps unsurprisingly the training of professionals in palliative care is also a relational endeavour and relies upon structured mechanisms for supervision and reflection and mutual support for colleagues.

## Discussion

Where principled approaches to clinical ethics focus on what ought to be done or decided, the starting point for an ethics of care is that we are in this together, in relationship, facing illness or death together. Decisions about what to say or do, and how to allow space for moral emotions, flow from that connection with one another. As such, there is a natural resonance between the philosophy of ethics of care and the inherent value of the relationships that healthcare practitioners in palliative care cultivate in their day-to-day practice. Particularly when a patient and their family are facing end of life care we recognise the importance, as described by Eva Feder Kittay, of “giving care and responding appropriately to care, empathy, and fellow feeling” [44]. We sought to evaluate from the literature how ethics of care applies to palliative care and found an overarching message that palliative care patients, their families and the professionals who care for them are crucially interlinked. This is the basic premise of ethics of care or relational ethics, that there is an ideal of human flourishing that is based upon a caring, interdependent, embodied, and socially connected picture of human nature [45]. We found that when applied to palliative care, relational ethics can help guide both ways of being with patients and families and ways of being with ourselves and each other on a palliative care team.

Contrary to critiques of ethics of care that it is insufficiently action-guiding [46], this scoping review has confirmed that it is able to offer useful guidance for palliative care practitioners to shape and enhance the care they deliver. In ethics of care, there is a recognition that to be compassionate to another we are required to empathise, to apprehend the other’s reality and to “feel what s/he feels as nearly as possible” [1]. This requires the one-caring to be receptive and responsive to the needs of the cared-for. This ‘being with responsiveness and respect’ emerged as important for compassionate palliative care practice which recognises vulnerability but simultaneously respects a patient’s autonomy. Similarly, ‘being interconnected with empathy’ can have profound benefits for patients and may improve care across both the adult and paediatric spectrum. Whilst ‘being’ in this sense is not necessarily an act of medical practice as traditionally conceived, we would argue that orientation towards others, attentiveness and the ability to hold space for others, particularly at times of immense suffering, is a core act of day-to-day palliative care practice. Much of the moral action here resides in the mental preparation and the mindful commitment to being present with patients and relatives who are approaching the end of their lives or who face existential distress.

Professionals often find hope to be a particularly challenging concept in palliative care and may struggle to reconcile the fact that hope, which is generally ‘future-orientated’, is seemingly incompatible with the obvious reality that palliative care patients have a necessarily limited future ahead of them [47]. Relational ethics can offer useful guidance here by giving professionals the ability to weigh up different considerations, such as the need for hope and a sense of empowerment against the need for honesty and compassion, within any particular given patient relationship. By focusing on curious and responsive relationships in palliative care, practitioners may also be able to uncover the breadth of things that their patients (and/or their relatives and carers) may be hoping for, such as hope for reconciliation, for symptom control or for the chance to make it home or to meet a particular relative [48].

Finally, the review brought to light the insights that the ethics of care can offer to palliative care practitioners when considering their relationship with themselves and the work that they do. The painful side of deep connection with patients and their families is sharing in the sadness and range of emotions that accompany a patient’s death. An ethics of care recognises distress as the rule rather than the exception, encouraging greater preparation and support within teams and hospitals for those providing palliative care. Integrating the fundamental principles from ethics of care into education and training for practitioners has the potential to improve self-awareness and strengthen compassion within care. The literature highlighted how, with adequate training and institutional support, an ethics of care approach may encourage professionals to find community and connection in this work, reflecting on the meaningfulness of their practice in a way that offers greater work satisfaction.

### Special relevance to paediatric palliative care

Whilst there were only a small number of papers which addressed the ethics of care in the context of paediatric palliative care, our review highlighted the special relevance to this sub-specialty. In paediatric palliative care, we are faced with the distinctive dynamics of the child/parent relationship which is embedded within a wider family network (incorporating siblings, grandparents etc.) and this makes the relational aspect of the ethics of care especially apt. There is a network of relationships acting in multiple directions– parent to child, parent to health care professional and child to health care professional– all of which are dependent on care. We have noted in this review a particular relevance for complex ethical dilemmas involving decision-making for critically unwell children and for considering how to ensure that children and adolescents have a voice in their care. By taking a relational approach which recognises how children and families are “always already entwined in relationships which embue their lives with meaning” [40], this may allow professionals to help families to make difficult decisions about a child’s care. Navigating hope within paediatric palliative care can be challenging for both parents and professionals [49] but trusting, compassionate relationships informed by an ethics of care can allow a space for both honesty and hope to coexist.

### Future directions

While the review demonstrates a promising conceptual case for a valuable connection between ethics of care and palliative care practice, further normative work is needed to fully articulate a palliative ethics of care. The few studies suggest the need for more empirical research, particularly in paediatric palliative care. We note that none of the studies we found addressed ethics of care in the context of perinatal palliative care. The theory may prove to be especially pertinent to this nascent subspecialty within paediatric palliative care where the interests of the fetus or newborn baby are deeply entwined with those of the parents, and expectant mothers experience unique vulnerabilities when receiving care [50, 51]. Finally, while the papers reviewed were from a range of countries, they reflected largely high-income settings and hospitals and lacked a more diverse cultural representation of care ethics in palliative care in, for example, African and Southeast Asian settings.

### Limitations

Our search strategy aimed to identify all relevant literature across multiple databases but there is a risk that some relevant papers may have been overlooked. We recognise that excluding papers which were not available in English introduces the potential for English-language bias and that we may have missed some valuable perspectives by doing so. We did not search the grey literature as we did not expect it would yield significant findings relevant to our topic of interest, but this may have resulted in missing pre-prints or patient stories published online. Given the heterogenous nature of the papers retrieved and the mix of conceptual or normative papers with empirical studies, we did not perform a critical appraisal of the papers for their methodological quality. In bioethics there is a recognised lack of suitable methods or criteria for the quality appraisal of normative literature [52].

We acknowledge the potential for bias particularly when interpreting conceptual papers and attempting to amalgamate ideas into discrete themes. We ensured that a second reviewer was consulted on which papers to include or exclude and was involved in the analysis and development of the themes. We also employed a constant comparative approach throughout the analysis with ongoing reflection and reflexivity to consider our own personal and professional perspectives as authors.

## Conclusions

The inherently emotional aspects of palliative care call for greater attention to the moral emotions arising in relationships with patients and families during a difficult and potentially deeply meaningful time. Emerging work suggests that an ethics of care offers useful conceptual and practical tools for informing practice, training, and ways of being with patients, families, and ourselves.

## Electronic supplementary material

Below is the link to the electronic supplementary material.

Supplementary Material 1

Supplementary Material 2

## Data Availability

The datasets used and/or analysed during the current study are available from the corresponding author on reasonable request.

## References

[1] Noddings N. Caring: a relational approach to ethics and moral education. University of California Press; 2013.

[2] Newnham E, Kirkham M. Beyond autonomy: care ethics for midwifery and the humanization of birth. Nurs Ethics. 2019;26:2147–57. 10.1177/0969733018819119 30638112 10.1177/0969733018819119

[3] Austin W, Bergum V, Dossetor J. Relational ethics: an action ethic as a foundation for health care. In: Tschudin V, editor. Approaches to ethics: nursing beyond boundaries. New York: Butterworth-Heinemann; 2003. pp. 45–52.

[4] World Health Organization. WHO Fact sheet: Palliative care. https://www.who.int/news-room/fact-sheets/detail/palliative-care (accessed 29 November 2024).

[5] Aghaei MH, Vanaki Z, Mohammadi E. Emotional bond: the nature of relationship in palliative care for Cancer patients. Indian J Palliat Care. 2020;26:86. 10.4103/IJPC.IJPC_181_19 32132791 10.4103/IJPC.IJPC_181_19 PMC7017707

[6] Norlock K. Feminist Ethics. In: Zalta EN, ed. The Stanford Encyclopedia of Philosophy (Summer 2019 Edition). Metaphysics Research Lab, Stanford University 2019. Available at: https://plato.stanford.edu/archives/sum2019/entries/feminism-ethics/ (Accessed 20 March 2025).

[7] Lindemann H. Feminist ethics of care and responsibility. In: Lindemann H, editor. An invitation to feminist ethics. Oxford University Press; 2019. p. 0.

[8] Rachels S, Rachels J. The elements of moral philosophy. 7th ed. New York: McGraw-Hill; 2012.

[9] Parsons JA, Johal HK. In defence of the bioethics scoping review: largely systematic literature reviewing with broad utility. Bioethics. 2022;36:423–33. 10.1111/bioe.12991 34969147 10.1111/bioe.12991

[10] Arksey H, O’Malley L. Scoping studies: towards a methodological framework. Int J Soc Res Methodol. 2005;8:19–32. 10.1080/1364557032000119616

[11] Gilligan C. In a different voice: psychological theory and women’s development. Harvard University Press; 1993.

[12] Aramesh K. Compassion as the reunion of feminine and masculine virtues in medicine. J Med Ethics Hist Med. 2017;10:8. 29296257 PMC5747837

[13] Bergman T, Lize N, Beijer S, et al. A care ethics approach to a reduced ability to eat. Nurs Ethics. 2024;31:420–31. 10.1177/09697330231197708 37767623 10.1177/09697330231197708 PMC11308360

[14] Berlin A. Close encounters of the first kind: an interdisciplinary ethics of care approach mitigates moral injury and family division in the midst of Covid-19. J Pain Symptom Manage. 2022;64:e159–64. 10.1016/j.jpainsymman.2021.03.028 34022390 10.1016/j.jpainsymman.2021.03.028 PMC9361467

[15] Borgstrom E, Walter T. Choice and compassion at the end of life: A critical analysis of recent english policy discourse. Soc Sci Med. 2015;136–137:99–105. 10.1016/j.socscimed.2015.05.013 10.1016/j.socscimed.2015.05.013 25989003

[16] Branch WT. A piece of my Mind. The ethics of patient care. JAMA. 2015;313:1421–2. 10.1001/jama.2015.1080 25871666 10.1001/jama.2015.1080

[17] De Panfilis L, Di Leo S, Peruselli C, et al. I go into crisis when… ethics of care and moral dilemmas in palliative care. BMC Palliat Care. 2019;18:1–8. 10.1186/s12904-019-0453-2 31399094 10.1186/s12904-019-0453-2 PMC6689155

[18] de Vries M, Leget CJW. Ethical dilemmas in elderly cancer patients: a perspective from the ethics of care. Clin Geriatr Med. 2012;28:93–104. 10.1016/j.cger.2011.10.004 22326037 10.1016/j.cger.2011.10.004

[19] Drongowski SH. Beyond ‘justice vs care’: Can the ethic of care coordinate with the ethic of justice to support palliative care?. UNLV Retrospective Theses & Dissertations. 1994;43.

[20] Gilbert R, Lillekroken D. Nurses’ perceptions of how their professional autonomy influences the moral dimension of end-of-life care to nursing home residents– a qualitative study. BMC Nurs. 2024;23:216. 10.1186/s12912-024-01865-5 38549064 10.1186/s12912-024-01865-5 PMC10976790

[21] Grealish L. Beyond Hippocrates: ethics in palliative care. Int J Palliat Nurs. 1997;3:151–5. 10.12968/ijpn.1997.3.3.151 29324101 10.12968/ijpn.1997.3.3.151

[22] Guité-Verret A, Vachon M, Girard D. Intentional presence and the accompaniment of dying patients. Med Health Care Philos. 2023;26:477–86. 10.1007/s11019-023-10161-z 37338776 10.1007/s11019-023-10161-z PMC10425290

[23] Ho CW-L, Lin C-C. How the COVID-19 pandemic could reshape palliative care into High-Tech and High-Touch care: an ethics of care perspective. Cancer Nurs. 2020;43:429–30. 10.1097/NCC.0000000000000883 33079793 10.1097/NCC.0000000000000883

[24] Hudson H, Wright DK. Towards a guiding framework for prison palliative care nursing ethics. Adv Nurs Sci. 2019;42:341–57. 10.1097/ANS.0000000000000266 10.1097/ANS.0000000000000266 30839333

[25] Leung D, Esplen M. Alleviating existential distress of cancer patients: can relational ethics guide clinicians? Eur J Cancer Care. 2010;19:30–8. 10.1111/j.1365-2354.2008.00969.x 10.1111/j.1365-2354.2008.00969.x 19912294

[26] Morberg Jämterud S. Acknowledging vulnerability in ethics of palliative care - A feminist ethics approach. Nurs Ethics. 2022;29:952–61. 10.1177/09697330211072361 35225042 10.1177/09697330211072361 PMC9289980

[27] Nogueira VP, Furtado MA, de Pessoa VLM. Ethical dilemmas at the end of life: a reflection from the philosophical perspective of Luigina Mortari. Rev Bras Enferm. 2023;76Suppl(3):e20220759. 10.1590/0034-7167-2022-0759 10.1590/0034-7167-2022-0759 PMC10695058 38055527

[28] Olsman E, Willems D, Leget C. Solicitude: balancing compassion and empowerment in a relational ethics of hope—an empirical-ethical study in palliative care. Med Health Care Philos. 2016;19:11–20. 10.1007/s11019-015-9642-9 25944316 10.1007/s11019-015-9642-9 PMC4805712

[29] Olthuis G, Dekkers W. Professional competence and palliative care: an ethical perspective. J Palliat Care. 2003;19:192–7. 14606332

[30] Pergert P, Lützén K. Balancing truth-telling in the preservation of hope: a relational ethics approach. Nurs Ethics. 2012;19:21–9. 10.1177/0969733011418551 22140184 10.1177/0969733011418551

[31] Peter E, Liaschenko J. Moral distress reexamined: A feminist interpretation of nurses’ identities, relationships, and responsibilites. J Bioethical Inq. 2013;10:337–45. 10.1007/s11673-013-9456-5 10.1007/s11673-013-9456-5 23754182

[32] Ramvi E, Ueland VI. Between the patient and the next of kin in end-of-life care: A critical study based on feminist theory. Nurs Ethics. 2019;26:201–11. 10.1177/0969733016688939 28116964 10.1177/0969733016688939

[33] Smith KA, Stajduhar K. Using relational ethics to approach equity in palliative care. Palliat Care Soc Pract. 2024;18:26323524241293820. 10.1177/26323524241293820 39525427 10.1177/26323524241293820 PMC11544665

[34] Wasson K. The ethics of care or the ethics of justice? A middle way. Open Univ. 1998. 10.21954/ou.ro.0000e179. PhD thesis.

[35] Wright D, Brajtman S, Bitzas V. Human relationships at the end of life: an ethical ontology for practice. J Hospice Palliat Nurs. 2009;11:219–29. 10.1097/njh.0b013e3181aada4c

[36] Brierley J, Larcher V. Cui Bono?? Can feminist ethics show a path in complex decision-making where ‘classical’ theories cannot? Clin Ethics. 2011;6:86–90. 10.1258/ce.2011.011013

[37] Moreton KL. Reflecting on ‘hannah’s choice’: using the ethics of care to justify child participation in end of life Decision-Making. Med Law Rev. 2020;28:124–54. 10.1093/medlaw/fwz011 31257451 10.1093/medlaw/fwz011

[38] Moreton KL. The ethics of care and healthcare decision-making involving children in mid-childhood. Ph.D.: University of Birmingham; 2017.

[39] Neefjes V. Parental ethical decision making and implications for advance care planning: A systematic review and secondary analysis of qualitative literature from England and Wales, Germany, and the Netherlands. J Palliat Med. 2023;26:1728–43. 10.1089/jpm.2022.0520 37262127 10.1089/jpm.2022.0520

[40] Walter JK, Ross LF. Relational autonomy: moving beyond the limits of isolated individualism. Pediatrics. 2014;133. 10.1542/peds.2013-3608D 10.1542/peds.2013-3608D 24488536

[41] Whitty-Rogers J, Alex M, MacDonald C, et al. Working with children in end-of-life decision making. Nurs Ethics. 2009;16:743–58. 10.1177/0969733009341910 19889915 10.1177/0969733009341910

[42] Silverman D. *Interpreting qualitative data.* 6th Edition. London: SAGE 2020.

[43] Held V. The ethics of care: personal, political, and global. USA: Oxford University Press; 2006.

[44] Kittay E. At the margins of moral personhood. Ethics. 2005;116:100–31. 10.1086/454366 16578953 10.1086/454366

[45] Groenhout RE. Connected lives: human nature and an ethics of care. Rowman & Littlefield; 2004.

[46] Slote M. Caring versus the philosophers. Selected essays. Oxford University PressNew York, NY 2009;260–70.

[47] Nekolaichuk CL, Bruera E. On the nature of hope in palliative care. J Palliat Care. 1998;14:36–42. 9575712

[48] Feudtner C. The breadth of hopes. N Engl J Med. 2009;361:2306–7. 10.1056/NEJMp0906516 20007559 10.1056/NEJMp0906516

[49] Bertaud S, Suleman M, Wilkinson D. Hope pluralism in antenatal palliative care. J Med Ethics Published Online First: 14 Oct. 2024. 10.1136/jme-2024-110120 10.1136/jme-2024-110120 PMC12322395 39401847

[50] Marty CM, Carter BS. Ethics and palliative care in the perinatal world. Seminars Fetal Neonatal Med. 2018;23:35–8. 10.1016/j.siny.2017.09.001 10.1016/j.siny.2017.09.001 28916237

[51] Jenkinson B, Kruske S, Kildea S. The experiences of women, midwives and obstetricians when women decline recommended maternity care: A feminist thematic analysis. Midwifery. 2017;52:1–10. 10.1016/J.MIDW.2017.05.006 28528239 10.1016/j.midw.2017.05.006

[52] Mertz M, Strech D, Kahrass H. What methods do reviews of normative ethics literature use for search, selection, analysis, and synthesis? In-depth results from a systematic review of reviews. Syst Reviews. 2017;6:261. 10.1186/s13643-017-0661-x 10.1186/s13643-017-0661-x PMC5738202 29258598

