# Correction: The heart of palliative care is relational: a scoping review of the ethics of care in palliative medicine

**DOI:** 10.1186/s12904-025-01818-y

**Published:** 2025-08-01

**Authors:** Sophie Bertaud, Dominic Wilkinson, Maureen Kelley

**Affiliations:** 1 Nuffield Department of Population Health, Ethox Centre, University of Oxford, Big Data Institute, Old Road Campus, Oxford, OX3 7LF UK; 2 Louis Dundas Centre for Children’s Palliative Care, Great Ormond Street Hospital for Children, London, UK; 3 Uehiro Oxford Institute, University of Oxford, Oxford, UK; 4 John Radcliffe Hospital, Oxford, UK; 5 Department of Internal Medicine, Wake Forest University School of Medicine, Winston- Salem, USA; 6 Center for Bioethics, Health & Society, Wake Forest University, Winston- Salem, USA

**Correction: BMC Palliat Care 24, 150 (2025).**

10.1186/s12904-025-01784-5.

Following publication of the original article [1], the reported that Table 2 caption should be updated from “Studies included in the review. Papers highlighted in green had a paediatric focus.” to “Studies included in the review.”

The original article [1] has been corrected.

## References

[1] Bertaud S, Wilkinson D, Kelley M. The heart of palliative care is relational: a scoping review of the ethics of care in palliative medicine. BMC Palliat Care. 2025;24:150. 10.1186/s12904-025-01784-5. 40420093 10.1186/s12904-025-01784-5 PMC12105298

