# Supplementary material for: The heart of palliative care is relational: a scoping review of the ethics of care in palliative medicine
Source: BMC Palliat Care. 2025 May 26;24:150. doi: 10.1186/s12904-025-01784-5 (PMC12105298; doi:10.1186/s12904-025-01784-5)
Supplement: Supplementary file 1 — Supplementary Material 1 [file 12904_2025_1784_MOESM1_ESM.docx]

**Supplementary File 1: Coding document**

Research question: How does ethics of care apply to palliative care (both adult and paediatric)?

Secondary research questions:

- How can ethics of care illuminate the key activities of palliative care practitioners?
- How can ethics of care guide and improve palliative care practice?
- What is the role of ethics of care in palliative care education and training?

| Code | Definition | Example quotes |
| --- | --- | --- |
| Attentiveness | - Paying close attention to one another, listening carefully. - The quality of attuning to and taking care of others. (Closely related to responsiveness.) | “We maintain that especially attentiveness and competence are caring virtues that should be fostered among healthcare professionals. Attentiveness particularly applies here to recognizing the psychosocial impact of reduced ability to eat.” (Bergman et al, 2024)  “For elderly cancer patients being attentive and responsive to the patient's perspective seems to be a major precondition for delivering good quality care attuned to the need, perspective and vulnerable position of patients” (De Vries & Leget, 2012) |
| Responsiveness | - The quality of reacting to a situation or person in a way that is aligned with what is needed. - Responding to the needs of others and responding in a timely manner. | “Put simply, the ethics of care require the application of digital health in ways that enhance crucial elements of care, including (a) attentiveness to the needs of patients and caregivers, (b) responsibility in roles of care, (c) competence in appropriate and abled use of digital technologies, and (d) responsiveness of the care receiver to digitally enhanced care.” (Ho and Lin, 2020) |
| Reciprocity | - Mutual dependence, action, or influence. - Two-way exchange. - Caring moral obligations based upon a notion of give and take. | “In accompaniment, we argue that a dynamic of reciprocity is initiated when the clinician witnesses and inherits the work that the patient accomplishes in the face of suffering and death.” (Guite-Verret et al, 2023) |
| Vulnerability | - The state of being unable or less able to protect one's own interests. - Being more susceptible to physical or psychological harm or exploitation. - People may be vulnerable when they are highly dependent on others to meet their needs. - (Meant to be balanced with respect and agency.) | “Inherent vulnerability, which underlines the self as situated in relation to others and dependent on others, can be combined with respect for autonomy in the form of relational autonomy” (Morberg Jämterud, 2022) |
| Autonomy | - The quality or state of being self-governing. - The right of patients to make informed decisions about their medical care. | “By contrast, taking a more relational approach to how individuals and families are always already entwined in relationships that imbue their lives with meaning leads providers to respect autonomy by helping families make decisions.” (Walter and Ross, 2014) |
| Connectedness/  interconnectedness | - The state of being joined or linked, connected with each other. - May include shared interests. - (Overlap with reciprocity.) | “(…) [R]elational ethics helps us to understand how the relationships of children are interconnected with their parents and health care providers.” (Whitty-Rogers et al, 2009)  “With the result that children would be recognized as being interconnected and benefiting from close relations with others—especially parents, and allow us to begin from a presumption of trust within those relationships.“ (Moreton, 2019) |
| Dependence/  interdependence | - The state of relying on someone or something else. - The idea that all people are dependent on others for care at some point in their lives. - Care ethics understands interdependence as a necessary aspect of the human condition. | “It provided a means to recognize the interdependence of the individual in a family, social and cultural group and was arguably better suited to the poignant dilemmas that may arise in critical care settings.“ (Brierley and Larcher, 2011) |
| Compassion/empathy | - Sympathetic consciousness of others' distress together with a desire to alleviate it. - Being aware of and sharing another person's feelings, experiences, and emotions. - Providing care through empathy, respect, and dignity. | “Finally, compassion can be called the merging and reunion point of the feminine and masculine virtues in pursuing the goals of healthcare and medicine. Developing and strengthening this virtue should be a part of both formal and hidden curriculums in medical education in every medical school all over the world.” (Aramesh, 2017) |
| Moral distress | - The psychological unease generated when professionals identify an ethically correct action to take but are constrained in their ability to take that action. - An emotional state of sadness, anxiety or regret following a difficult decision or dilemma where all options were non-optimal. | “We argue that moral distress is the response to constraints experienced by nurses to their moral identities, responsibilities, and relationships.” (Peter and Liaschenko, 2013) |
| Health care professional well-being | - Ability to be comfortable, healthy or happy in one’s work. - A combination of physical, mental, and emotional health, and satisfaction with one‘s career. | “We argue that fundamental principles are relational and involve ideas of relational ethics. These themes guide an approach that can better meet patients’ and families’ needs, as well as impact on the work satisfaction of clinicians.” (Leung & Esplen, 2008) |
| Complex decision-making | - The process of making choices when there are multiple factors, uncertainties, and stakeholders involved. | “Clearly, much more is involved than decisions about whether and when to stop treatment and what is ‘fair’, and/or whose decision it is. What is equally important is the intricate and intimate web of relationships between and among children, families, and those who become deeply involved with them in times of profound life significance.” (Whitty-Rogers et al, 2009) |
| Conflict resolution | - The process of finding a peaceful solution to a disagreement between two or more parties. | “This case highlights the seemingly impossible conundrums facing the paediatric intensive care team, yet reveals how moral thinking outside the restrictions of more traditional current medical ethics may help ease moral dilemmas and lead to a resolution that recognizes and supports the humanity of those involved.” (Brierley and Larcher, 2011) |
| Key activities of palliative care | - What must palliative care health care practitioners do to succeed in their role? - What does good palliative care look like? | “Attentiveness particularly applies here to recognizing the psychosocial impact of reduced ability to eat and the tense situations that may arise from this between the patient and family members, whereas competence applies to knowing how to deal well with these tensions, while attending to the care needs of both the patients and the family.” (Bergman et al, 2024) |
| Enhancing, guiding or improving practice | - How can we improve the experience of palliative care provision? (for patients, families and HCPs) | “If we should attempt to suggest implications for nursing practice from this study, we would emphasise the significance of having a manager that may facilitate systematic supervision where ethical reflections and awareness of nurses’ ‘use of self’ in their practice is in focus.” (Ramvi & Ueland, 2019) |
| Education and training | - Acquiring knowledge and learning how to do something as a medical practitioner. | “Our conclusion points up the importance of education in ethics in the development of professional competence, and argues that because palliative care involves intense human interactions, integrating palliative care into the medical curriculum may improve the ethical culture of health care as a whole.” (Olthuis & Dekkers, 2003) |
